# Contactless Capacitive Electrocardiography Using Hybrid Flexible Printed Electrodes

**DOI:** 10.3390/s20185156

**Published:** 2020-09-10

**Authors:** Mathieu Lessard-Tremblay, Joshua Weeks, Laura Morelli, Glenn Cowan, Ghyslain Gagnon, Ricardo J. Zednik

**Affiliations:** 1 École de Technologie Supérieure (ÉTS), Université du Québec, Montréal, QC H3C 1K3, Canada; mathieu.lessard-tremblay.1@ens.etsmtl.ca (M.L.-T.); laura.morelli.1@ens.etsmtl.ca (L.M.); ghyslain.gagnon@etsmtl.ca (G.G.); 2 SIG.NUM Preemptive Healthcare, Inc., Montréal, QC H3K 1G6, Canada; jweeks@sig-num.com; 3 Department of Electrical and Computer Engineering, Concordia University, Montréal, QC H3G 1M8, Canada; gcowan@ece.concordia.ca

**Keywords:** flexible hybrid electronics, electrode design, capacitive electrocardiography, printed electronics, wearables

## Abstract

Traditional capacitive electrocardiogram (cECG) electrodes suffer from limited patient comfort, difficulty of disinfection and low signal-to-noise ratio in addition to the challenge of integrating them in wearables. A novel hybrid flexible cECG electrode was developed that offers high versatility in the integration method, is well suited for large-scale manufacturing, is easy to disinfect in clinical settings and exhibits better performance over a comparable rigid contactless electrode. The novel flexible electrode meets the frequency requirement for clinically important QRS complex detection (0.67–5 Hz) and its performance is improved over rigid contactless electrode across all measured metrics as it maintains lower cut-off frequency, higher source capacitance and higher pass-band gain when characterized over a wide spectrum of patient morphologies. The results presented in this article suggest that the novel flexible electrode could be used in a medical device for cECG acquisition and medical diagnosis. The novel design proves also to be less sensitive to motion than a reference rigid electrode. We therefore anticipate it can represent an important step towards improving the repeatability of cECG methods while requiring less post-processing. This would help making cECG a viable method for remote cardiac health monitoring.

## 1. Introduction

Traditional electrocardiographic (ECG) measurement systems that rely on contact electrodes (electrodes which form a galvanic/ohmic connection with the patient’s body) present challenges when ECG monitoring is required immediately, unobtrusively and frequently. Traditional adhesive contact electrodes require placement by a trained healthcare provider on a clean, prepared skin surface to ensure accurate location (and therefore morphology) and signal quality [1]. Limitations of standard wet gel contact electrode placement include placing them on the body correctly and removing them within their time limit to avoid skin reactions [2].

Capacitive ECG (cECG) technology is a potential alternative to the century-old traditional contact-based ECG technology and promises to revolutionize many aspects of ECG acquisition in both home and professional healthcare environments. cECG eliminates the need for trained professionals to perform the ECG acquisition on patients by being able to operate through clothing and on unprepared skin. cECG acquisition through clothing does not cause skin irritation and therefore does not have an application time limit, thus facilitating long-term, continuous monitoring.

cECG can be accomplished by capacitively coupling biopotential amplifiers to the patient [3]. Typical source capacitances can be on the order of 1–100 pF, producing source impedances of up to approximately 1 TΩ for frequencies of interest (0.05–150 Hz). Maintaining constant signal gain at such low frequencies is the major challenge in designing a cECG acquisition system. Feasibility of such amplifiers has been proven [4] and non-linear bias networks can provide a method for transient suppression [5]. Furthermore, the option offered by capacitive electrodes to acquire a signal through clothing and to integrate these electrodes in an array of sensors opens many possibilities. Hence, it becomes possible to sense the cardiac biopotentials with an unprecedently large number of sensors. Combined with the advances in artificial intelligence, this paves the way for automated analysis and diagnostics of the signals. It represents an important step towards the democratization of the cECG as we anticipate the era where such systems can be used widely for remote monitoring of the patients [6].

While wet electrodes provide a low-impedance electrical connection, contactless electrodes must tolerate a high impedance capacitive coupling of the cardiac biopotentials [7]. In both cases, the electrode bandwidth must be at least as large as the bandwidth of the frequency content in Q, R and S waves that compose the QRS segment of the cardiogram which is normally between 0.67 Hz and 5 Hz [8]. An electrical model of capacitive electrode for ECG acquisition is described and analyzed under the Electrical model section.

Reported by Leonhardt et al., several cECG systems have been integrated in car seats, beds, chairs, bathtubs and even toilet seats [9]. However, the different options available present either poor signal-to-noise ratio [10,11,12], limited comfort, challenges for manufacturing at large scale or a difficult integration into small systems (e.g., wearables or medical equipment) without significant design changes.

Previous research in hybrid flexible electronics for cECG applications indicates that one critical challenge that causes low signal-to-noise ratio in a capacitive system is that it relies on the patient’s body to conform to the electrode to produce an equivalent source capacitance for all the system’s channels [11,13]. Lee J. et al. [11] highlight that some areas of the body tend to conform better than others to flat surfaces, introducing high coupling variability between the electrode and the patient’s skin. To overcome this limitation, the authors introduce a conductive foam to limit the air gap between the patient skin and the electrode. Poliks et al. [14] improve the flexibility and the comfort of the electrode by printing a sensing surface and soldering small surface mounted components onto a flexible polyimide substrate. The electrode they developed is not capacitive, but they proved that it was possible to use a printed flexible substrate as a support for an ECG acquisition device.

We address the need for a capacitive cECG sensor that is flexible, efficient and easy to integrate in various devices. A novel Flexible Hybrid Electronics electrode was developed leveraging the strengths of both classical Printed Circuit Board (PCB) and printed electronics. The novel flexible electrode consists of a conductive ink surface that is printed on a 30 mm x 30 mm polyester substrate. A regular PCB populated with surface mount electronic components is attached to the underside of the substrate.

The electrode implemented using the novel method is flexible and can conform to a minimal bending diameter of 75 mm. It offers a consistent mismatch in low frequency cut-off for a range of different body shapes and meets minimal frequency requirements with measured values between 210 and 475 mHz. The novel flexible electrode, by comparison to the state of the art rigid electrodes, is less sensitive to large amplitude motion artifacts, such as respiratory activity, due to its low cut-off frequency variability. Its flexibility and simple construction makes it convenient to integrate into different types of devices.

## 2. Electrical Model

**Figure 1:**
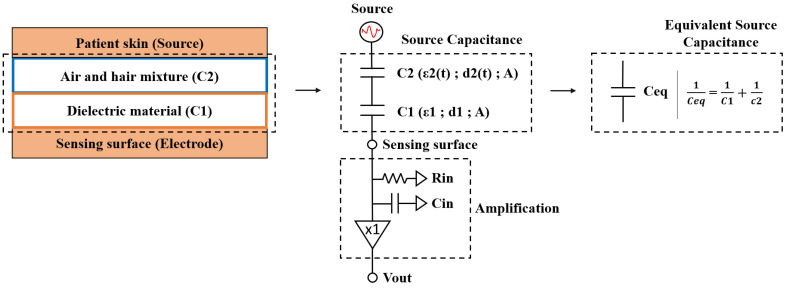
Capacitive coupling of skin biopotentials and electrode.

A simplified electrical model of the electrode coupling to the patient skin is presented in Figure 1. In the case of a cECG electrode, the capacitance of the coupling between the electrode and the skin is the source capacitance. The input impedance of the preamplifier is on the order of 1 TΩ within the measured frequencies (0.1–200 Hz).

The mid-band gain ($A_{m}$) of the system is:

(1) $$A_{m}=\frac{C_{eq}}{C_{eq}+C_{in}}$$

where $C_{in}$ is the preamplifier input capacitance and $C_{eq}$ is the equivalent source capacitance.

The frequency content of the Q, R and S cardiac waves that compose the QRS cardiac segment, important for clinical diagnosis, is within the 0.67 Hz to 5 Hz range [8]. The frequency at which the system gain decreases by 3 dB relative to the pass-band gain is defined as the low frequency cut-off. This value is governed by the capacitive coupling of the electrode to the patient as it forms a first-order high-pass filter with the input impedance of the preamplifier. Therefore, the low-frequency cutoff pole resulting from the source capacitance must be lower than 0.67 Hz. In most applications, Ceq >> Cin. Therefore, the cut-off frequency ($f_{c}$) of the cECG electrode can be simplified to [11]:

(2) $$f_{c}=\frac{1}{2\pi R_{in}C_{eq}}$$

where $R_{in}$ is the amplifier input resistance.

The source capacitance depends on effective electrode geometry and is similar to a parallel plate capacitor [11]. It is defined by the following parameters: capacitance (C), area of the plate (A), distance (d), relative permittivity of the dielectric ($\varepsilon_{r}$), permittivity of free space ($\varepsilon_{0}$).

(3) $$C=\frac{\varepsilon_{0}\varepsilon_{r}A}{d}$$

Decreasing the distance between the plates increases the capacitance and lowers the low-frequency cut-off. It is therefore necessary to clearly understand the impact coming from the patient’s morphological variations on the pass-band gain and cut-off values.

**Figure 2:**
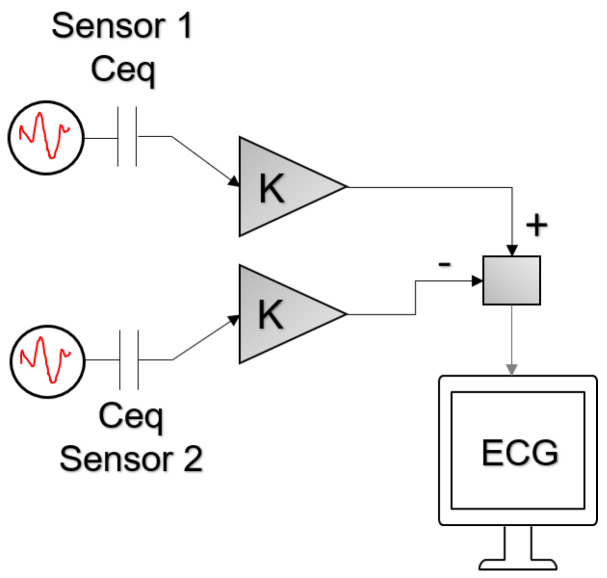
Differential signal path of the skin biopotentials to the display.

Most ECG systems rely on differential signals, which are obtained by the subtraction of one signal in a location from a second signal in another location. The differential signal pathway of the biopotentials capacitively coupled to the electrode is presented in Figure 2. With equal gains (K) along both channels, noise that is common to both sensors can be cancelled by subtracting one channel’s signal from the second one.

However, to ensure efficient common-mode rejection, the system requires source capacitance and cut-off frequencies that are consistent among the input channels. Minimizing the mismatch of measured source capacitance and low frequency cut-off is therefore an effective way to minimize the noise at the output of the differential signal.

**Figure 3:**
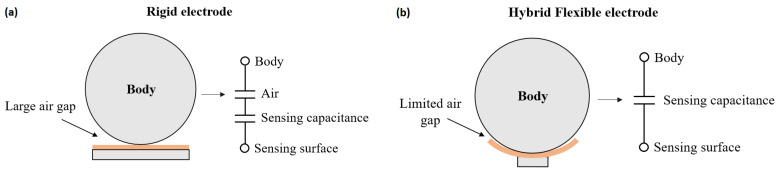
Schematized system geometry for: (**a**) Rigid electrode. (**b**) Hybrid Flexible electrode.

As modeled in Figure 3, it is expected that a flexible electrode will be more consistent in term of source capacitance by conforming to the patient’s body more evenly on every channel, reducing the distance variation between the body and the electrodes (Figure 3b).

## 3. Electrode Design

The novel electrode uses a Flexible Hybrid Electronics design, combining both classical and printed electronics features and components. A cECG preamplifier designed using conventional electronics manufacturing processes is integrated onto the flexible electrode substrate. Signals coupled to the sensing surface are amplified by the preamplifier before being transmitted to a remote device using conductive traces protected by a dielectric coating.

**Figure 4:**
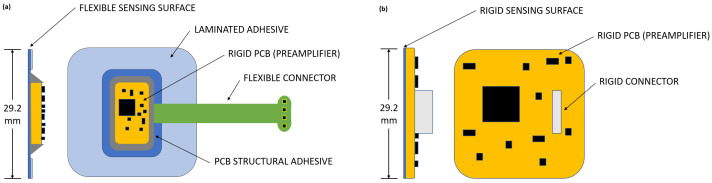
Component description of electrodes: (**a**) Flexible hybrid electrode proposed. (**b**) Reference rigid electrode.

**Figure 5:**
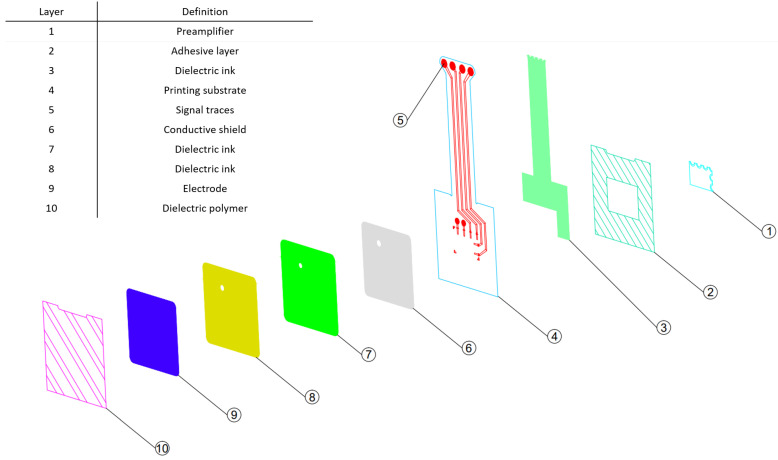
Hybrid flexible electrode-layer breakdown.

A rigid electrode was similarly designed to isolate the effect of the flexible electrode mechanically conforming to the body. To allow direct comparison between electrodes, both electrodes share the same amplification stage and sensing surface area. Schematics of both the flexible and the rigid electrodes are presented in Figure 4:

A detailed layer breakdown of the novel flexible electrode is described in Figure 5. The novel flexible electrode structure is composed of several conductive layers (6, 9) and dielectric layers (7, 8), printed on a polyester substrate (4). A Polyimide layer (10) is laminated on the top of the stack using structural adhesives to protect the flexible circuit layers and prevent conductive coupling of the electrode with the patient when the electrode assembly is physically contacting the skin.

A cECG preamplifier (1), is integrated on the flexible electrode polyester substrate (4). Signals coupled to the sensing surface (9) are amplified by the preamplifier before being transmitted to a remote device using conductive traces (5) protected by a dielectric coating (3). The same amplifier circuit is used in the rigid reference electrode to allow for a fair comparison of the flexible sensing surface with the reference rigid surface. Finally, another layer of structural adhesive is laminated on the bottom of the electrode (2) to provide a means to integrate the electrode assembly in a sensor array embedded in a hospital grade mattress.

The choice of hybrid design offers the combined advantages of printed electronics and classical PCB design with off-the-shelf components. As shown further in the results section, a flexible sensing surface conforms better to the sensed surface and gives an enhanced performance when comparing with the rigid sensing surface; both flexible and rigid contactless electrodes compared favorably to traditional contact ECG performance. Additionally, by using a regular PCB to hold standard surface components we obtain a preamplifier stage that can be combined easily with future iterations on the flexible part of the electrode. Alternatively, it would have also been possible to print an array of independent electrodes, each with independent preamplifiers, that share a common upper dielectric film to improve integration capabilities.

Special care was given to the selection of low temperature curing inks and soldering processes. This gives more options for future iterations of the printing substrate as high temperature stability will not be a requirement.

## 4. Experimental Setup

To assess the effect of electrode flexibility on performance, the sensing area, dielectric layer and amplification stages for both the proposed flexible electrode and the reference rigid electrode are the same.

**Figure 6:**
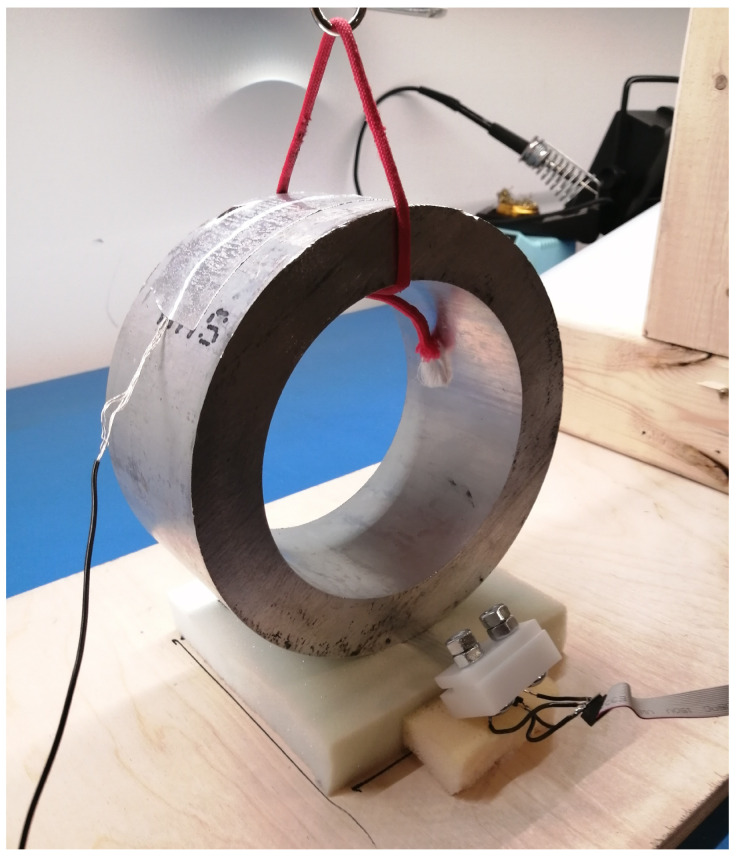
Setup for pass-band gain and cut-off frequency acquisition.

Three metrics are used to measure the performance: cut-off frequency, pass-band gain and source capacitance. A test apparatus shown in Figure 6 was developed to simulate capacitive coupling with a patient’s body. It is composed of a weighted aluminum cylinder suspended from a beam positioned over the electrode. The electrode is placed with the sensing surface up on a foam pad in order to mimic as much as possible a real clinical test. Cylinders of various diameters, ranging from 3” to 12” were used to simulate the curvature of typical body parts that electrodes would be positioned against during ECG acquisition. The force exerted onto the cylinder over the electrode is maintained at 20N during all the tests.

A dynamic signal analyzer (model SR780 by Stanford Research Systems) is used to measure the electrode voltage transfer function by applying a known signal to the aluminum cylinder and measuring the output of the electrode preamplifier. The cut-off frequency and pass-band gain are measured from the transfer function.

The pass-band gain is defined as the gain measured at 200 Hz. Then, to measure the cut-off frequency, the SR780 analyzer is configured with the parameters presented in Table 1.

The source impedance is measured directly using a Stanford Research Systems model SR715 RLC meter. It is used in 4-point probe mode for source capacitance measurement. The preamplifier is disconnected from the electrode and capacitance between the aluminum cylinder and the electrode impedance is measured.

## 5. Results

**Figure 7:**
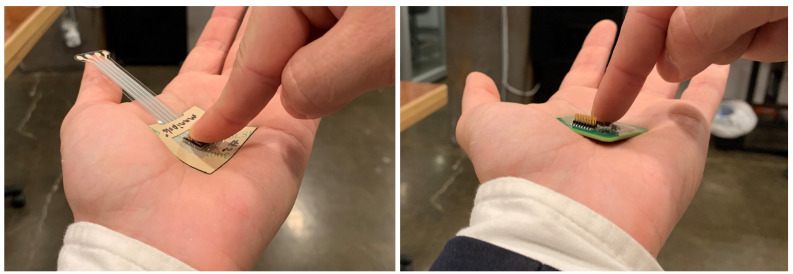
Electrode conformability comparison: (**left**) hybrid flexible electrode. (**right**) Rigid electrode.

Mechanically, the flexible electrode requires minimal force to conform to a curved surface whereas the rigid reference sensor does not conform appreciably, even with large forces exerted on it, as illustrated in Figure 7. The flexible sensor thereby avoids the sharp edges of a rigid sensor that could be uncomfortable and "dig into" the patient; however, both rigid and flexible sensors do not require an ohmic contact adhering to the naked skin surface, so either contactless option can be assumed to be more comfortable than traditional ECG electrodes. Nevertheless, additional, long-term tests would be necessary to quantify the improvement in patient comfort.

**Figure 8:**
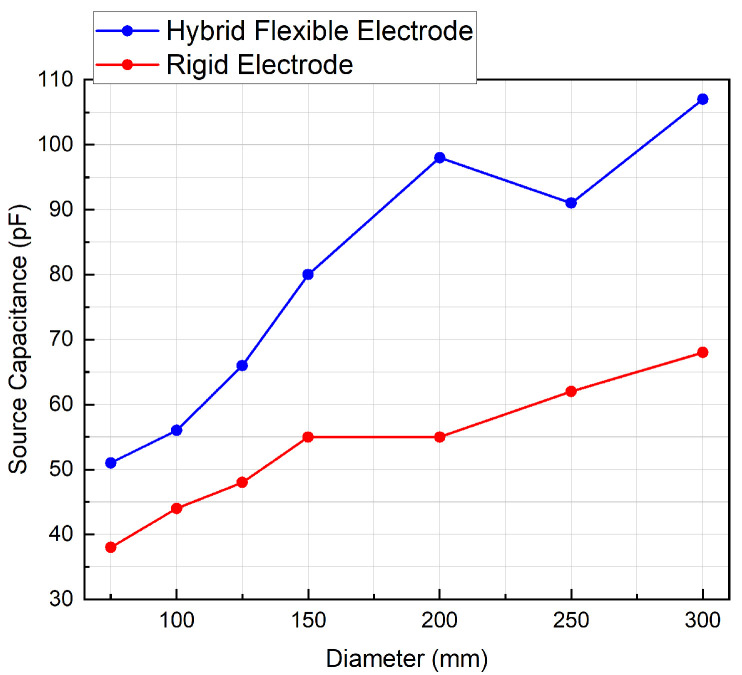
Capacitance vs. body diameter for hybrid flexible vs. rigid electrode.

As a consequence of its flexibility, the novel flexible electrode exhibits a greater capacitive coupling with the cylindrical test surfaces than the rigid electrode, for all tested cylinder diameters, as illustrated in Figure 8.

The effect of the gap tends to decrease with decreasing cylinder diameter, as the hybrid flexible sensor reaches its minimal bending diameter which is estimated at 75 mm. From that point, an air gap is always present between the electrode and the cylinder when reducing the diameter, so the value of capacitance decreases drastically as can be expected.

**Figure 9:**
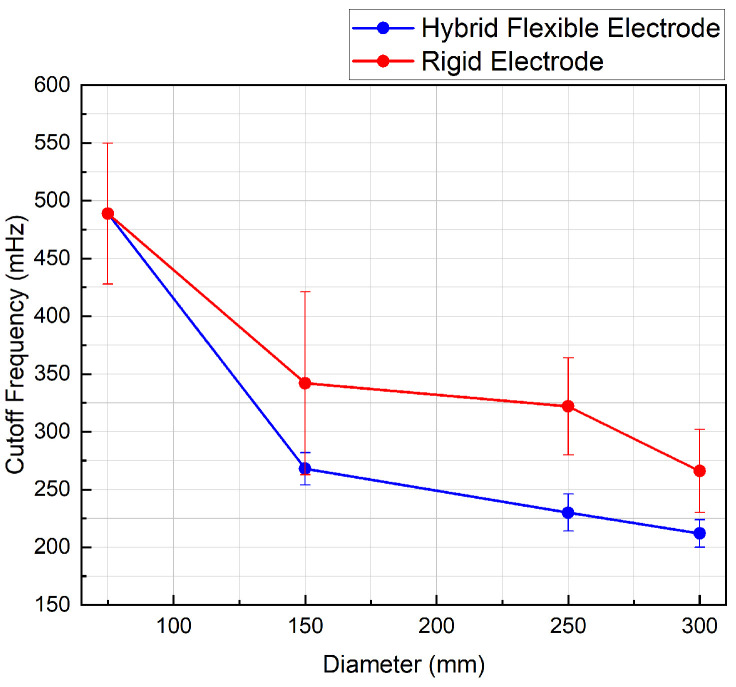
Average cut-off frequency vs. body diameter for hybrid flexible and rigid electrodes. Average measurements over 5 samples of each electrode type.

As shown in Figure 9, the novel flexible electrode has a lower average cut-off frequency than the rigid reference electrode.

The cut-off frequency of the hybrid flexible electrodes exhibits generally less variation than the rigid sensors until it reach its minimal bending radius. Across all measurements, the standard deviation among samples is reduced by 78% using the flexible sensor.

**Figure 10:**
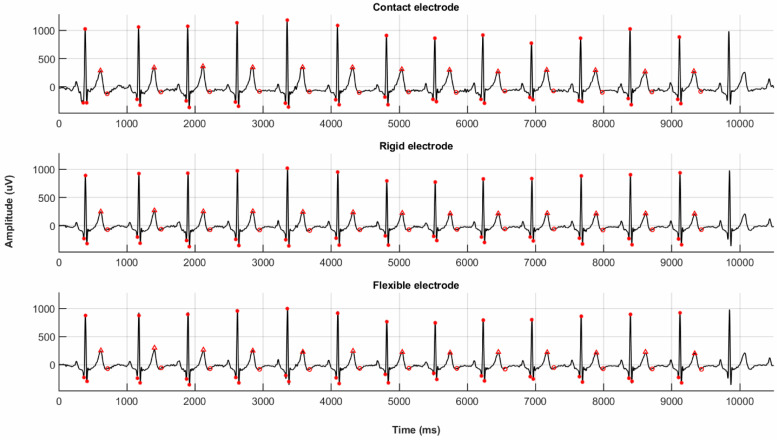
ECG spectra obtained simultaneously for these three acquisition methods: (**top**) Contact electrode. (**middle**) Rigid electrode. (**bottom**) Hybrid flexible electrode.

Finally, a cECG was acquired using the novel flexible and rigid electrodes, as well as a traditional clinical 3M red dot electrode using arm limb lead positions according to the Student Health Center Manuals [2]. Figure 10 shows the resulting ECG spectra obtained simultaneously for these three acquisition methods.

The measurements were performed simultaneously on the same healthy 27 year old male patient. The ECG spectra obtained using the novel flexible and rigid electrodes are almost indistinguishable from those obtained using traditional clinical contact electrodes. This is further confirmed when comparing extracted ECG metrics, including the voltage peaks and durations for Q, R, S and T waves, as shown in Table 2: the quantitative results suggest that the novel flexible and rigid electrodes perform comparably to traditional contact electrodes while avoiding the necessity of an ohmic adhesive contact.

To define the ECG signal to noise ratio (SNR), a pass band method was used. The power spectral density between 5 and 40 Hz is the signal (S) and the power spectral density outside from these boundaries is the noise (N). The values for signal and noise are then input in Equation (4).

(4) $$SNR=10log_{10}\frac{S}{N}$$

The flexible electrode, rigid electrode, and traditional clinical contact electrode exhibit a very similar noise level and QRS timing. In the cECG acquired with the novel flexible and rigid electrodes, it is easy to recognize the crucial QRS segments used for medical diagnosis. The fact that these features are easily identified even without the implementation of any baseline wander filtering for these two novel prototypes illustrates the efficiency of its common-mode noise reduction, and shows that they compare favorably to state-of-the art traditional ECG leads currently used in clinical settings.

## 6. Discussion

Measurements for the novel flexible electrode show that the source capacitance is significantly higher than a rigid electrode when taking into account various patients morphology. Both the flexible and rigid electrodes meet the specified frequency requirements (0.67–5 Hz), but the lower variation in cut-off frequency across tested cylinder diameters of the flexible electrode implies better channel-to-channel matching.

In the context of cECG acquisition, a better channel-to-channel matching in the pass-band gain between electrodes produces less common-mode to differential-mode conversion in the cECG signals. The performance of the flexible sensor is better than the rigid one because of the better common-noise reduction at low frequencies and improved capacitance (requiring less post-processing). Otherwise either cECG electrode compares favorably to traditional state-of-the-art clinically employed contact electrodes.

Better performance in terms of repeatability and uniformity of critical metrics such as pass-band gain and low frequency cut-off means less active filtering is needed to remove unwanted information from the ECG spectrum. As a result, less filtering implies that the ECG is less susceptible to data attenuation within the low frequency range [15,16].

Respiratory activities involve frequency content in the range of 0.12 Hz to 0.5 Hz [8]. This happens to be within the measured cut-off frequency range for the flexible and the rigid electrode (0.2 to 0.5 Hz—Figure 9). Because of the efficiency of its common-mode conversion, it is expected that the novel flexible electrode will not be very sensitive to large amplitude motions such as those caused by respiratory activity. Further testing on a large number of human participants and devices will be needed to confirm this intuition.

## 7. Conclusions

The mechanical and electrical performance evaluation suggests that a flexible capacitive electrode could be a good candidate for large adoption for two main reasons: the use of a flexible sensing surface improves the signal quality at low frequency and makes it easy to integrate the flexible electrode into arrays with curved or soft surfaces.

Low frequency cut-off and pass-band gain values of the flexible electrode are appropriate for medically important QRS detection and shown to be superior to a rigid electrode. In addition, the novel flexible electrode is expected to be less sensitive to large amplitude motion artifacts such as respiratory activity due to its low cut-off frequency variability within the frequency range of human respiration. When compared to state-of-the-art contact electrodes, the SNR of the capacitive electrodes (rigid or flexible) are similar. Timing of the QRS are similar as well, so cECG is not a disruptive technology in terms of SNR, but when leveraging a flexible sensing surface it is a promising new technology in terms of easier mechanical integration and potentially improved comfort, while maintaining simple signal fitering (and therefore more efficient and accessible circuit design). It promises to provide the same reliable ECG signal currently required by clinicians without requiring the adhesion of uncomforlocationcontact electrodes, although a full range of clinical tests is required to confirm this potential.

Although the novel flexible electrode presented in this study is merely a first prototype, we have confirmed in principle that flexible electrode should be explored for use in contactless ECG systems, as they promise improved signal quality over a comparable rigid sensor while providing sufficient mechanical flexibility for integration into long duration monitoring solutions and wearables. In addition, preliminary testing shows that both the novel flexible electrode and the novel rigid electrode perform no worse than the established, state-of-the-art, adhesive contact electrodes currently used in clinical settings. Although these exciting results are very promising, a clinical trial using both healthy individuals and patients suffering from cardiovascular diseases is necessary to evaluate the medical diagnostic value.

## Figures and Tables

**Table 1:** SR780 analyzer measurement parameters for the electrode’s voltage transfer function.

Parameter Name	Value
Signal type	Swept sine
Starting frequency (mHz)	200
Frequency step (mHz)	2
Ending frequency (mHz)	600
Amplitude (mV)	860

**Table 2:** Extracted parameters from electrocardiogram (ECG) and contactless electrocardiogram (cECG) simultaneous acquisition: signal-to-noise ratio (SNR), QRS and QT amplitude and duration.

Electrode	SNR	QRS Amplitude (μV)	QRS Duration (ms)	QT Duration (ms)
Contact	12.59	1217	47	275
Rigid	14.31	1117	44	275
Flexible	13.71	1084	45	275
